# LPI Radar Detection Based on Deep Learning Approach with Periodic Autocorrelation Function

**DOI:** 10.3390/s23208564

**Published:** 2023-10-18

**Authors:** Do-Hyun Park, Min-Wook Jeon, Da-Min Shin, Hyoung-Nam Kim

**Affiliations:** Department of Electronics Engineering, Pusan National University, Busan 46241, Republic of Korea; dohpark@pusan.ac.kr (D.-H.P.); gow1128@pusan.ac.kr (M.-W.J.); ekals2020@pusan.ac.kr (D.-M.S.)

**Keywords:** electronic warfare, low-probability-of-intercept, signal detection, deep learning, time-series analysis

## Abstract

In electronic warfare systems, detecting low-probability-of-intercept (LPI) radar signals poses a significant challenge due to the signal power being lower than the noise power. Techniques using statistical or deep learning models have been proposed for detecting low-power signals. However, as these methods overlook the inherent characteristics of radar signals, they possess limitations in radar signal detection performance. We introduce a deep learning-based detection model that capitalizes on the periodicity characteristic of radar signals. The periodic autocorrelation function (PACF) is an effective time-series data analysis method to capture the pulse repetition characteristic in the intercepted signal. Our detection model extracts radar signal features from PACF and then detects the signal using a neural network employing long short-term memory to effectively process time-series features. The simulation results show that our detection model outperforms existing deep learning-based models that use conventional autocorrelation function or spectrogram as an input. Furthermore, the robust feature extraction technique allows our proposed model to achieve high performance even with a shallow neural network architecture and provides a lighter model than existing models.

## 1. Introduction

With the increasing complexity of electronic warfare, the superiority of friendly forces is considerably influenced by the electronic warfare support (ES) system capabilities. These ES systems are tasked with detecting, identifying, and locating the sources of radiated emissions from adversaries, thereby recognizing threats and influencing contemporary warfare strategies [1]. The effectiveness of ES systems depends on their ability to detect enemy radar signals and extract information accurately. However, the emergence of low-probability-of-intercept (LPI) radar signals has increased detection difficulty. LPI radars incorporate characteristics such as wideband modulation and frequency agility, making detection by adversarial ES systems more challenging. Among these characteristics, the low power transmission is the most challenging, making LPI radar signals difficult to detect. Owing to the low power broadcasting of LPI radars, the signal-to-noise ratio (SNR) of the signals intercepted by ES systems is below 0 dB, substantially complicating detection [2]. The lack of a reliable detection algorithm for LPI radars can lead to strategic disadvantages and potential detriment.

Over the past several decades, considerable effort has been expended in research to detect radar signals. This includes techniques using energy as a test statistic for signal detection [3], methods employing frequency domain analysis [4,5], and method utilizing visibility graphs [6]. These methods have been primarily used as algorithms for radar signal detection due to their effectiveness. However, these conventional methods often fail when facing the low signal power and frequency agility of LPI radar signals.

Recently, deep learning algorithms based on convolutional neural networks (CNNs) or recurrent neural networks (RNNs) have demonstrated considerable potential in signal processing. Deep learning algorithms are also being applied in radar signal processing. Moreover, deep learning-based signal detection techniques have shown superior detection performance compared to conventional methods [7,8,9].

While existing deep learning-based algorithms have shown effective performance in signal detection, these methods rely on feature extraction techniques that do not consider the unique characteristics of radar signals, leading to performance limitations in radar signal detection. These existing methods often struggle to adapt to the dynamic and complex characteristics of radar signals, making it a challenge to achieve optimal detection performance. Therefore, using feature extraction techniques tailored for radar signals allows for overcoming the limitations in detection performance. This adaptation enables the extraction of more relevant and distinct features, enhancing the detection accuracy and efficiency. We propose a robust detection method by leveraging feature extraction techniques that utilize the periodicity characteristic of radar signals. Considering the pulse repetition characteristic of radar signals, we utilized the periodic autocorrelation function (PACF) to capture and analyze periodic signals and provide richer information. Additionally, our model employs radar signal detection neural networks structured with long short-term memory (LSTM), demonstrating high efficacy in processing time-series features. The primary objective of this study is to contribute to ES system advancement by introducing a robust method for LPI radar signal detection. This paper demonstrates that integrating PACF and deep learning approaches can substantially enhance the detection capabilities of ES systems compared to existing algorithms and solve the challenges raised by LPI radar signals.

The remainder of this paper is organized as follows. In Section 2, we introduce the LPI radar signals received by the ES system and present previous studies related to signal detection. Section 3 provides a detailed explanation of the proposed LPI radar signal detection method. In Section 4, we present the simulation environment and performance analysis results. In addition, we highlight the detection capabilities of our proposed method by comparing it with existing deep learning-based detection algorithms. Finally, the discussions and future research directions are presented in Section 5, and the conclusions are summarized in Section 6.

## 2. Research Background

The signal detection algorithm is central in the ES system and allows a quick understanding of the enemy’s location, activities, and tactics. However, inaccurate detections can result in strategic warfare setbacks, emphasizing the demand for rapid and precise signal detection algorithm development. In this section, we discuss the LPI radar signals received by the ES system and the methods for detecting these signals.

### 2.1. LPI Radar Signal

Signals intercepted by the ES system include the transmitted radar signals x(n) and white Gaussian noise w(n). Intercepted signal y(n) can be represented as follows:

(1) $$y\left(n\right)=x\left(n\right)+w\left(n\right)=A\exp\left(j2\pi f\left(n\right)\left(nT_{s}\right)+\phi \left(n\right)\right)+w\left(n\right),$$

where *n* is the sample index increasing every $T_{s}$ for a sampling frequency $f_{s}$, *A* is the amplitude of the radar signal, and f(n) and ϕ(n) indicate the frequency and phase modulation functions, respectively. Radar modulation improves target detection resolution, reduces interference, and optimizes performance across diverse environments and conditions. Therefore, the selection of f(n) and ϕ(n) can determine the modulation technique used. One of the key modulation schemes used in radar is linear frequency modulation (LFM), which improves range resolution in target detection by exhibiting a linearly changing frequency within a pulse [10]. Another modulation scheme using the frequency modulation function is the Costas code, which features frequency hopping to reduce interference in radar systems [11]. In contrast, Barker code modulates the phase by shifting it using binary code sequences designed to have low autocorrelation properties [2]. The frequency and phase modulation functions for the three modulation signals considered in our study are presented in Table 1. *B* represents the modulation bandwidth of LFM, $\tau_{pw}$ denotes the pulse width, and $f_{i}$ represents the Costas sequence.

### 2.2. Signal Detection

The primary focus of this study is to determine whether a signal exists in the intercepted signal y(n). Therefore, we consider the following binary hypothesis testing:

(2) $$\begin{array}{cc} \; & H_{0}:y\left(n\right)=w\left(n\right),\\ \; & H_{1}:y\left(n\right)=x\left(n\right)+w\left(n\right). \end{array}$$

Here, the null hypothesis $H_{0}$ indicates no signal within the intercepted signal. The alternative hypothesis $H_{1}$ signifies a radar signal within the intercepted signal. To determine which of the two hypotheses to accept, we generate a test statistic T(y) from the intercepted signal and compare it to a pre-established threshold value λ. This process of hypothesis selection can be represented as follows:

(3) $$\begin{array}{cc} \; & T\left(y\right)<\lambda \to H_{0},\\ \; & T\left(y\right)\geq \lambda \to H_{1}. \end{array}$$

Extensive research has been conducted on generating test statistics that can effectively detect signals. Among these developed techniques, the energy detection method has been widely employed to detect the signal by employing the energy of the received signal as the test statistic [3]. These energy detectors offer implementation simplicity and effective operation regardless of the modulation scheme. However, they are sensitive to changes in noise levels and exhibit poor performance in low SNR environments. Detection techniques that utilize frequency domain analysis to generate test statistics for signal detection have been developed to address noise issues. For example, an entropy detector [4] uses the uncertainty in the frequency domain for signal detection, considering that the randomness of frequency components is proportional to entropy. The method uses statistical estimation through weighted sum operations between each frequency component and its corresponding probability values. Another frequency analysis detection technique [5] leverages the theoretical property that higher-order statistical moments are zero when the probability density function is Gaussian. However, detection techniques based on frequency domain analysis may encounter performance degradation. These techniques perform well when energy is concentrated in a single frequency component, as with unmodulated signals or Barker codes. However, problems arise when energy is spread out in the frequency domain, as with LFM and Costas code.

Recently, deep learning techniques have gained significant attention as powerful tools in signal processing. Particularly, their application to signal detection techniques has demonstrated remarkable performance improvements. Recent techniques for signal detection use short-time Fourier transform (STFT) and CNN [8]. These approaches transform the received signal into images using time-frequency analysis to apply image classification techniques commonly employed in computer vision. Furthermore, signal detection techniques that combine autocorrelation functions (ACFs) and one-dimensional CNN [9] have been proposed. This approach uses the ACF’s capability to capture patterns and structures within time-series data. Experiments conducted in various SNR environments showed that these deep learning-based methods exhibited superior performance compared to conventional signal detection algorithms.

## 3. Proposed Signal Detection Method

**Figure 1:**
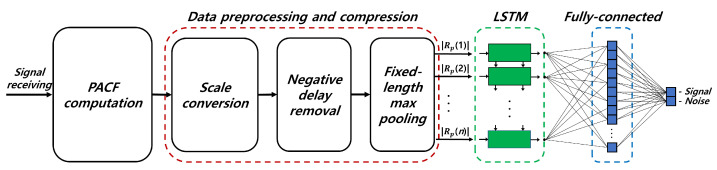
Block diagram of the proposed signal detection method.

Although existing signal detection techniques have contributed significantly to the advancement of signal detection, the demand for methods that offer improved accuracy, inference speed, and generalization capabilities persists. This section introduces a novel deep learning-based signal detection method that utilizes the inherent periodicity of radar signals and leverages periodicity analysis to achieve superior signal detection performance compared to conventional methods. Furthermore, by exploiting the periodicity feature of signals for signal detection, our approach exhibits generalization capabilities, allowing accurate detection of signals not included in the training dataset. In addition, the proposed model applies preprocessing steps that compress the data size while minimizing performance degradation for rapid signal detection. The overall block diagram of the proposed model is shown in Figure 1.

As shown in Figure 1, the proposed detection technique consists of the following steps: (1) Computation of PACF; (2) Data preprocessing and compression; (3) Feature extraction using LSTM network; (4) Signal detection using fully-connected network.

### 3.1. Periodicity Analysis

In this subsection, we introduce the ACFs used to generate the data input for the neural network employed for signal detection. Because the signals intercepted by the ES system are time-series data, these received signals can be used directly as input for the neural network. However, ES systems utilize a very high sampling frequency to receive signals, resulting in many samples in the intercepted signals. Hence, using the received signals directly as input data for the neural network can increase the computational demand for signal detection. Therefore, rather than using the received data directly as neural network input, extracting the features from the received signal and using them as the input proves effective for neural network training. Therefore, we introduce ACF, traditionally used for periodicity analysis, and PACF, which effectively captures the inherent periodicity features of the intercepted radar signals, in our proposed method.

#### 3.1.1. Autocorrelation Function

Time-series data shows that the current state’s value is closely related to past and future values. Such time-series data is said to have autocorrelation. ACF represents the degree of correlation over time and assesses the correlation between signals y(t) and y(t+τ), where y(t+τ) is y(t) shifted by a delay τ. The ACF exhibits significant values when the original signal components persist over time at a specific time delay τ. The ACF R(τ) for the received signal y(t) is expressed as follows:

(4) $$R\left(\tau \right)=\int_{-\infty }^{\infty }y\left(t\right)y^{*}\left(t+\tau \right)dt.$$

**Figure 2:**
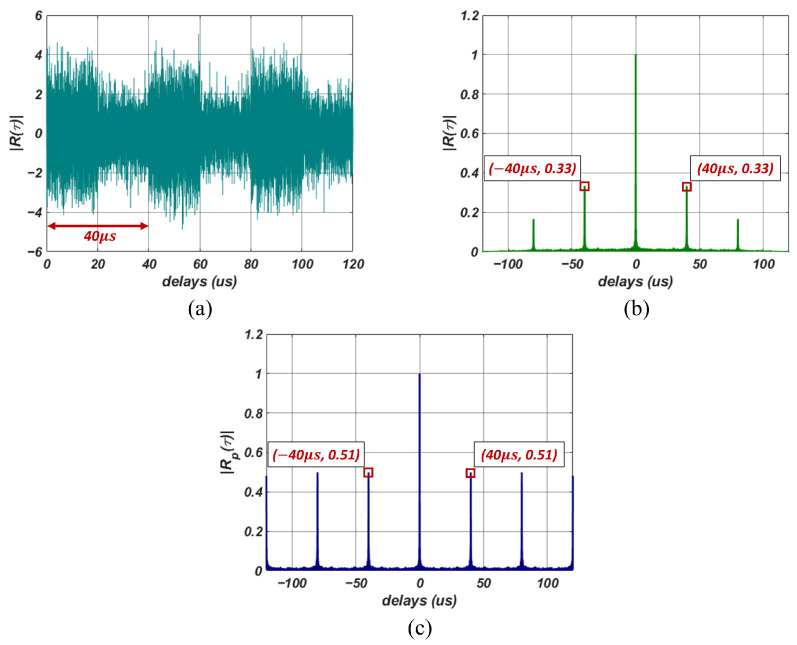
Examples of simulated radar signals and corresponding computed autocorrelation functions (ACFs). (**a**) Simulated LFM radar signal, (**b**) Computed ACF, (**c**) Computed PACF.

Figure 2a is an example of an LFM signal used to examine ACF. The simulated signal is a periodic signal with SNR of 0 dB with a signal acquisition time (*T*) of 120 μs, a pulse width ($\tau_{pw}$) of 20 μs, and a duty cycle of 50%, resulting in a pulse repetition interval ($T_{PRI}$) of 40 μs. The ACF computed using the signal in Figure 2a is shown in Figure 2b. The highest correlation is present at a time delay of 0 μs in the ACF. Additionally, due to the periodicity of the signal, we observe peaks at ±40 μs and ±80 μs in the ACF.

#### 3.1.2. Periodic Autocorrelation Function

In time-series data, the current state’s value is closely related to past and future values. In particular, when considering radar signals received by ES systems, they exhibit a distinct pulse repetition interval, representing a periodic signal where pulses repeat. For such radar signals, ACF depicts the degree of correlation over time. However, with increasing delay, there is a corresponding decrease in correlation, as shown in Figure 2b. The signal perfectly matches itself at τ=0, resulting in maximum ACF. However, as τ approaches signal acquisition time, only the correlation between the end and beginning of the signal is computed. Thus, only a portion of the signal matches, decreasing the ACF values. The issue of decreasing correlation with increasing delay, even when using periodic signals, makes it challenging to extract signal features in weak signal environments.

We utilize a modified ACF to overcome the issue of decreasing correlation with increasing delay. The modified ACF uses the received signal repetitively when calculating the correlation. We utilize an extended signal $y_{ext}\left(t\right)$ as follows, repeating y(t) when *t* exceeds a multiple of signal acquisition time *T*.

(5) $$y_{ext}\left(t\right)=y\left(t\;\mathrm{mod}\;T\right).$$

Using the extended signal, we compute PACF $R_{p}\left(\tau \right)$ as follows:

(6) $$R_{p}\left(\tau \right)=\int_{-\infty }^{\infty }y_{ext}\left(t\right)y_{ext}^{*}\left(t+\tau \right)dt.$$

Figure 2c shows the results of calculating PACF using simulated signals from Figure 2a. Compared to the ACF results in Figure 2b, the correlations at ±40 μs delay times are more pronounced in the PACF. Furthermore, the correlations at ±80 μs delays in the ACF exhibit relatively lower values, whereas in the PACF, they maintain substantial correlation values similar to those observed at ±40 μs delay times.

### 3.2. Data Preprocessing and Compression

**Figure 3:**
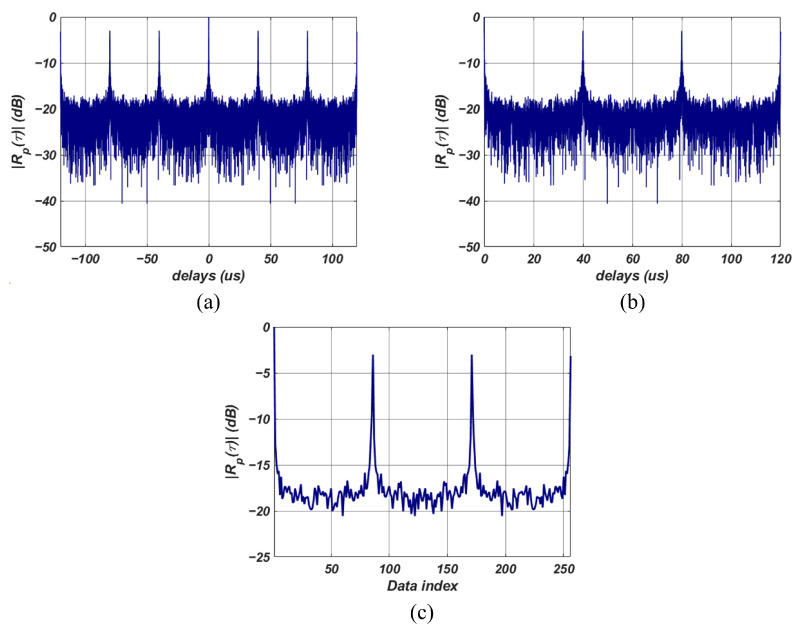
Results of the preprocessing steps. (**a**) decibel scale conversion process results, (**b**) negative delay removal process results, (**c**) fixed-length max pooling results.

Using PACF, we can accurately observe the periodic characteristics of radar signals, even in weak signal environments. To efficiently train the neural network and reduce computational load, we introduce a data preprocessing step before using PACF results in the neural networks. The data preprocessing consists of three steps: a unit conversion step, negative delay value removal step, and data compression step. The unit conversion step involves converting the PACF data, which has a linear scale, into a decibel scale to increase the learning efficiency of the deep learning model. Figure 3a shows the result of converting the PACF from Figure 2c. The peaks due to correlations are observed when examining the effects in Figure 2c with a linear scale. However, by converting the results into a decibel scale as shown in Figure 3a, we can observe the peaks caused by correlations and assess the sidelobe levels generated by the signal and noise.

The second data preprocessing step involves removing data related to negative delays and eliminating unnecessary data to reduce the computational load of the neural network model. PACF used for time-series analysis has a symmetric property around zero delay, as described by the following equation:

(7) $$R_{p}\left(\tau \right)=R_{p}\left(-\tau \right).$$

Negative delay values are redundant to perform signal detection with the data corresponding to positive delays. Therefore, when inputting PACF data into the neural network model, unnecessary negative delay values can be removed. This helps reduce the computational load of the neural network. Figure 3b shows the result of retaining only the data corresponding to positive delays from the PACF data in Figure 3a.

The final step in the preprocessing stage involves compressing the data using fixed-length max pooling. Max pooling is a commonly used technique in deep learning models to reduce data size [12]. It involves extracting the maximum value within a window and discarding the other values. Thus, the data size is reduced while preserving its inherent features, decreasing the computational load while minimizing performance degradation. The max pooling used in our proposed detector is configured with a variable window length, ensuring a predetermined fixed-length max pooling result. The fixed-length max pooling ensures that the neural network used for detection has a consistent computational load regardless of the signal’s length. Figure 3c displays the result of applying fixed-length max pooling to the PACF data from Figure 3b. In this example, the window length was set to achieve a data length of 256 after the pooling process. Applying the fixed-length max pooling technique highlights the peaks due to correlation while reducing the PACF data length.

### 3.3. Signal Detection Neural Network

#### 3.3.1. Long Short-Term Memory

In this subsection, we introduce LSTM, a type of RNN used in our proposed method to detect signals from the extracted PACF feature data. Unlike basic artificial neural networks (ANNs), RNN has a loop structure and contains hidden states within the network. The loop structure allows RNN to store information from past input data in hidden states and utilizes it for current and future data to improve the performance of neural networks. Due to these characteristics, RNN is known to be more efficient than traditional ANN in solving problems related to time-series data, such as natural language processing, video analysis, and signal classification [13,14,15,16].

**Figure 4:**
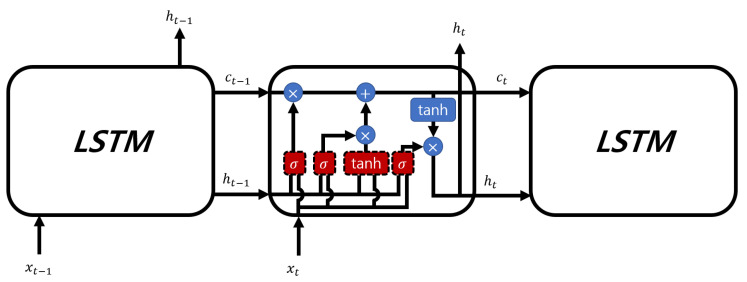
Architecture of the LSTM network.

A common issue with traditional RNN structures is the lack of long-term dependencies. In other words, these networks primarily rely on recent input data, and data from a distant past have limited influence on the processing of current input data. LSTM [17] was introduced to address this lack of long-term dependencies problem in standard RNNs. The key innovation of LSTM is utilizing the cell state to address the lack of long-term dependencies. The cell state is represented by the horizontal line at the top of the LSTM module, as shown in Figure 4.

The cell state is updated from the previous cell state value $c_{t-1}$ using simple linear operations to produce the current cell state $c_{t}$. LSTM calculates the forget gate output $f_{t}$, the cell state update value $i_{t}$, the new cell state candidates ${\tilde{c}}_{t}$, the new cell state $c_{t}$, the output gate value $o_{t}$, and the current hidden state $h_{t}$ as follows:

(8) $$\begin{array}{cc} \; & f_{t}=\sigma \left(W_{f}\cdot x_{t}+U_{f}\cdot h_{t-1}+b_{f}\right),\\ \; & i_{t}=\sigma \left(W_{i}\cdot x_{t}+U_{i}\cdot h_{t-1}+b_{i}\right),\\ \; & \tilde{c_{t}}=\tanh\left(W_{c}\cdot x_{t}+U_{c}\cdot h_{t-1}+b_{c}\right),\\ \; & c_{t}=f_{t}\circ c_{t-1}+i_{t}\circ \tilde{c_{t}},\\ \; & o_{t}=\sigma \left(W_{o}\cdot x_{t}+U_{o}\cdot h_{t-1}+b_{o}\right),\\ \; & h_{t}=o_{t}\circ \tanh\left(c_{t}\right), \end{array}$$

where σ(x) represents the sigmoid function; $x_{t}$ is the current input; $h_{t-1}$ is the previous hidden state; $U_{f}$, $W_{f}$, and $b_{f}$ are the weights and biases in the forget gate; $U_{i}$, $W_{i}$, and $b_{i}$ are the weights and biases in the input gate; $U_{c}$, $W_{c}$, and $b_{c}$ are the weights and biases when calculating the cell state candidates; $c_{t-1}$ represents the previous cell state; ∘ denotes Hadamard product; and $U_{o}$, $W_{o}$, and $b_{o}$ represent the weights and biases used to compute $o_{t}$.

#### 3.3.2. Fully-Connected Network

Our proposed signal detection technique employs a fully-connected network to determine the presence of a signal based on the extracted feature vectors from LSTM. The fully-connected network consists of an input and output layer. The input layer is composed of input nodes, each with a length equal to the number of hidden units in the LSTM. The output layer has two output nodes to calculate the probabilities associated with the presence or absence of a signal. Subsequently, the detection model applies the softmax function [18] to the values output from the two output nodes. The softmax function transforms the probabilities associated with the two nodes in the fully-connected network output layer. We employ the result of the softmax function from the node corresponding to the presence of a signal as a test statistic for signal detection. The threshold value for a specific false alarm rate has been established using the Neyman–Pearson criterion. This criterion aids in establishing a threshold that maximizes the detection probability for a given false alarm probability. Lastly, we can predict the presence of a radar signal by comparing the established threshold with the test statistic, as indicated in (3).

## 4. Detection Performance Analysis

### 4.1. Model Training

For training the detection neural network, we generated a training dataset consisting of three modulation schemes with various modulation parameters for each scheme. The parameters for the training dataset are summarized in Table 2.

The generated training dataset consists of 60,000 signals, which includes 30,000 signals with additive white Gaussian noise and 30,000 white Gaussian noise signals used for generating noisy signals. The 60,000 signals for the training dataset are transformed into input data using ACFs and the data preprocessing steps introduced in Section 3. Within the preprocessing steps, the ACFs generated from the signals are compressed to a length of 256 through fixed-length max pooling. The number of hidden units in the LSTM layer of the proposed detection model is 32. During the neural network training using the generated training dataset, the Adam optimization algorithm [19] was employed to update the weights of the LSTM and fully-connected network in the detection model. Through iterative experimentation, the hyperparameters that enable the proposed model to achieve the global minima were selected, and the hyperparameters used for model training are tabulated in Table 3. In the training process, the weights were updated using the 60,000 training data for 30 epochs. Additionally, the model was configured to learn rapidly with a high learning rate at the initial stage of training and more finely with a reduced learning rate in the later stages to converge to the optimal parameters. This was achieved by reducing the learning rate at a fixed ratio per every epoch.

### 4.2. Simulation Results

We analyze the comparative performance between the proposed detection model and existing signal detection methods. Within the realm of non-neural network detection methods, we considered an entropy detector, which uses the entropy of the received signal as a test statistic [4], and detector that utilizes the average degree of the visibility graph as a test statistic [6]. As introduced in Section 2, the deep learning-based algorithms considered for comparison include the STFT-CNN model, which converts intercepted signals into time-frequency images using the STFT and then utilizes a CNN for signal detection. Additionally, we considered an ACF-1DCNN model that analyzes correlation using ACF and performs signal detection using a 1-dimensional CNN. Calculating the neural network computational complexity in terms of floating point operations per second (FLOPS) for each detection neural network reveals that STFT-CNN has approximately 16.8 MFLOPS, ACF-1DCNN has 1.1 MFLOPS, and our proposed method, PACF-LSTM has 37.2 KFLOPS. Our proposed method exhibits the lowest computational complexity among these detection neural networks.

**Figure 5:**
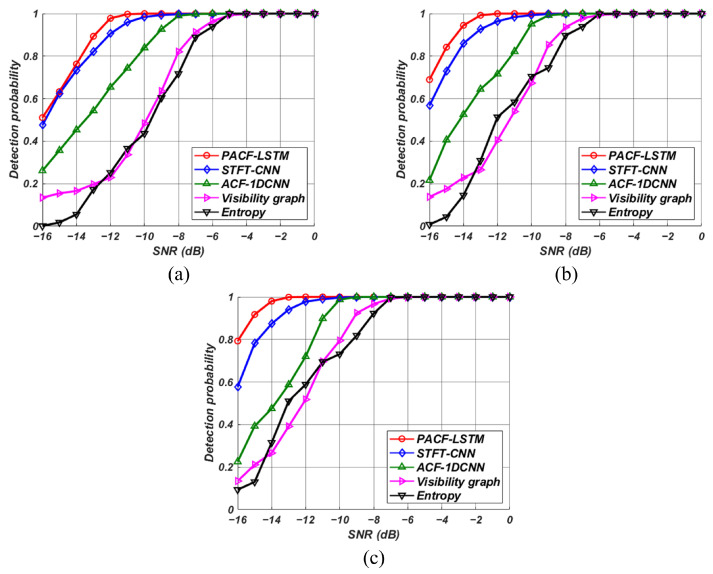
Detection probabilities of the detectors at various SNRs. Pulse width of (**a**) 10 μs, (**b**) 20 μs, and (**c**) 30 μs.

The test dataset used for performance comparison was constructed by generating 500 signals for each modulation scheme at a single SNR. The modulation parameters used to generate test signals were the same as those used in the training dataset, as shown in Table 2, with a fixed duty cycle of 50%. Figure 5 shows the detection probabilities of a detector with a false alarm probability of 0.1 for different SNRs across various pulse widths. All detectors demonstrated similar detection performance across all modulation schemes, and the results shown in Figure 5 represent the average detection probabilities for simulated signals with three different modulation schemes.

The detection performance analysis reveals that the proposed technique exhibits superior signal detection performance. Among the techniques considered, the STFT-CNN model exhibited the best performance, followed by the ACF-1DCNN model and non-neural network detection methods. Moreover, we confirmed that our proposed technique outperforms existing deep learning-based algorithms in weak signal detection performance. In the comparative analysis between the STFT-CNN, distinguished for its superior detection capability among existing methods, and our proposed model, it was found that the performance deviation was insubstantial at a pulse width of 10 μs. However, a significant performance discrepancy was observed as the pulse width increased. This can be attributed to a wider pulse width containing more energy in the radar signals, leading to more significant peak values from the correlation. Consequently, the proposed technique facilitates effective signal detection even in low SNR environments. Additionally, using time-series data analyzed with PACF rather than with ACF allows for superior detection performance even with neural networks having reduced computational complexity.

## 5. Discussion

We proposed a deep learning-based radar signal detection model that utilizes LSTM and PACF for periodicity analysis. Our model employed PACF as a time-series data analysis method, which provides rich information such as periodicity and sidelobe levels. The detection model used an LSTM to detect signals from feature data converted with various preprocessing steps applied to the computation results of PACF. Performance analysis using various modulation schemes revealed that the proposed model has remarkable detection capabilities. The analysis results demonstrated that the proposed detection method outperforms existing deep learning-based models. Furthermore, the proposed technique exhibited the best detection performance and achieved the lightest model compared to existing deep learning-based models. These results could be attributed to the powerful time-series data analysis technique employing PACF and preprocessing methods. Our proposed model achieved high performance even with considerably fewer neural network layers by extracting distinct and feature-rich input data with reduced size from intercepted signals. Therefore, the proposed method is a promising candidate for practical detection algorithms in ES systems requiring accurate and fast signal detection techniques. However, the proposed method presents a challenge for real-time implementation as it requires a sufficient signal acquisition time. Therefore, in the future, we plan to research on detection model that can be implemented in real-time using time-series analysis with PACF.

## 6. Conclusions

We introduced a model for detecting radar signals that exploit deep learning techniques and PACF for periodicity analysis. The model outperformed existing deep learning models in terms of detection probability and computational complexity. Therefore, we expect our proposed detection method to be effectively applied to ES systems. We plan to further research and develop a real-time detection model using time-series analysis with PACF.

## Figures and Tables

**Table 1:** Frequency and phase modulation functions for three modulation schemes.

Modulation Scheme	f(n) (Hz)	ϕ(n) (rad)
LFM	$f_{0}+B/\tau_{pw}\left(nT_{s}\right)$	constant
Costas code	$f_{i}$	constant
Barker code	constant	0 or π

**Table 2:** Parameters for training dataset generation.

Modulation Scheme	Parameter	Value
	Sampling frequency	50 MHz
	SNR	U(−20,10) dB
All	Pulse width	U(5,30) μs
	Duty cycle	U(25,100)%
	Signal acquisition time	$U\left(1,3\right)T_{PRI}$
LFM	Center frequency	U(6.25,12.5) MHz
	Modulation bandwidth	U(5,12.5) MHz
	Fundamental frequency	U(2.5,5) MHz
Costas code	Number of frequency hops	{4,6}
	Frequency spacing	U(2,4) MHz
	Center frequency	U(6.25,12.5) MHz
Barker code	Barker code length	{7,11,13}
	Cycles per phase code	{20,24,28}

U(·,·) denotes a uniform distribution.

**Table 3:** Hyperparameter used for training proposed signal detection model.

Hyperparameter	Value
Initial learn rate	$1\times 10^{-4}$
Learning rate reduction	3% per epoch
Epochs	30
Mini batch size	64
Input data length	256
Number of hidden units	32

## Data Availability

Not applicable.
